# DNA Damage Across Dietary Patterns: A Comet Assay Study in Vegans and Omnivores

**DOI:** 10.3390/foods15091477

**Published:** 2026-04-23

**Authors:** Ines Peremin, Marko Gerić, Ivone Jakasa, Goran Gajski

**Affiliations:** 1 Laboratory for Analytical Chemistry, Department of Chemistry and Biochemistry, Faculty of Food Technology and Biotechnology, University of Zagreb, 10000 Zagreb, Croatia; iperemin@pbf.hr (I.P.);; 2 Division of Toxicology, Institute for Medical Research and Occupational Health, 10000 Zagreb, Croatia

**Keywords:** vegan diet, omnivorous diet, DNA damage, DNA strand breaks, comet assay, cross-sectional study, human biomonitoring

## Abstract

Plant-based diets are generally associated with a reduced risk of chronic diseases; however, the relationship between a vegan diet and genome integrity remains insufficiently characterized. In this cross-sectional study, we assessed primary DNA damage in peripheral blood cells of vegans and omnivores. A total of 62 apparently healthy adults were included: 31 vegans (median vegan diet duration 4.5 years) and 31 omnivores matched for sex and smoking status. DNA damage was assessed using the alkaline comet assay under standardized conditions and expressed as tail intensity (% tail DNA), tail length, tail moment, and total comet area. Tail intensity was significantly higher in vegans than in omnivores (*B* = 1.98; 95% CI 0.19 to 3.76; *p* = 0.031) after adjustment for age, physical activity, body mass index (BMI), and alcohol consumption. Within the vegan group, longer duration of adherence to a vegan diet was positively associated with tail intensity, independent of age (*B* = 0.23; 95% CI 0.03 to 0.43; *p* = 0.026). These findings suggest that adherence to a vegan diet and its duration may be associated with higher levels of primary DNA damage; however, these results should be interpreted with caution given the observational design and modest sample size.

## 1. Introduction

A vegan diet represents one of the most restrictive forms of vegetarian dietary patterns, as it excludes all foods of animal origin, including meat, fish, dairy products, and eggs [1,2]. Differences in nutrient intake and bioavailability between vegan and omnivorous dietary patterns have been reported, particularly for micronutrients predominantly derived from animal sources [3,4,5]. Such variation in dietary composition may be associated with differences in physiological processes relevant to health, including those related to genomic stability [5,6,7,8].

DNA damage is widely regarded as one of the earliest events in the development of chronic diseases, cancer, and ageing [9,10,11,12,13]. Lifestyle factors, including diet and physical activity, have been associated with genomic stability, with proposed mechanisms involving oxidative stress, inflammation, and DNA repair processes [14,15]. Plant-based foods contain numerous bioactive compounds that may be involved in these pathways, and dietary intervention studies involving specific plant foods have demonstrated reductions in biomarkers of DNA damage and changes in DNA repair capacity [16,17,18,19,20,21]. Despite these potential protective effects, studies examining the relationship between vegetarian dietary patterns and genomic stability have produced inconsistent findings, as summarized in our systematic review [14].

In our previous studies, we observed differences in biomarkers of DNA damage between individuals adhering to traditional mixed and vegetarian diets. Vegetarians exhibited higher levels of chromosomal damage reflected by increased micronuclei frequency and DNA strand breaks [22]. More recently, we also observed lower primary DNA damage among omnivorous and pescatarian females compared with vegetarians [23]. Together, our findings suggest that dietary patterns may be associated with biomarkers of DNA damage.

Epidemiological studies suggest that higher levels of primary DNA damage measured by the comet assay are associated with increased risk of adverse health outcomes, highlighting the relevance of this biomarker for studies of DNA damage and related aspects of genomic stability [9,24]. However, direct comparisons of primary DNA damage between vegans and omnivores using the comet assay remain scarce. In the present study, we aimed to assess these differences using the alkaline comet assay.

## 2. Materials and Methods

### 2.1. Study Sample and Participant Selection

The cross-sectional study (*n* = 62) included 31 vegans and 31 omnivores recruited through voluntary response and snowball sampling from the local vegan community and the general population of Zagreb, Croatia. Recruitment was conducted using multiple channels, including social networks, social media, and public advertisements. All participants provided written informed consent and completed a structured questionnaire covering demographic characteristics, lifestyle factors (including physical activity, smoking, and alcohol consumption), medical history, and prior exposures relevant to the study outcomes. Vegan participants also reported their primary motivation for adopting a vegan diet as part of the questionnaire.

Vegan status was defined by self-reported exclusion of all animal-derived foods, as confirmed by a non-quantitative Food Frequency Questionnaire (FFQ) assessing habitual consumption frequencies over the preceding year. Participants in the vegan group reported habitual adherence to their respective diet for at least one year prior to enrolment. Omnivorous status was defined by habitual consumption of meat (including red meat, poultry, or processed meat), as assessed by the FFQ. Participants reporting consumption of fish or seafood but no meat (pescatarians) were excluded. Participants were individually matched 1:1 by sex and smoking status, with additional balancing by age (mean absolute within-pair difference 1.8 ± 1.5 years; range 0–7 years).

At the time of interview and blood collection, all participants were free of acute illness or infection, as indicated by high-sensitivity C-reactive protein (hs-CRP) concentrations < 10 mg/L in all cases. Medical conditions were recorded and categorized; however, none met the predefined exclusion criteria targeting active or severe conditions likely to influence systemic DNA damage, including diabetes, cardiovascular or neurodegenerative diseases, and other chronic inflammatory conditions with substantial systemic effects. Participants with a history of malignant tumors were excluded. Individuals with a body mass index (BMI) ≥ 30 kg/m^2^ were excluded due to obesity. Medication use at the time of sampling was documented, and individuals using systemic corticosteroids or other immunosuppressive therapy were excluded. Participants also reported no history of unprotected occupational exposure to known genotoxic or cytotoxic agents and no recent exposure to high-dose diagnostic ionizing radiation.

The study protocol was approved by the Ethics Committee of the Institute for Medical Research and Occupational Health, Zagreb, Croatia, the Ethics Committee of the University of Zagreb School of Medicine, and the study was conducted in accordance with the Declaration of Helsinki and applicable data protection regulations.

### 2.2. Lifestyle Assessment

Leisure-time physical activity was assessed using the Godin Leisure-Time Exercise Questionnaire (GLTEQ) [25]. A total weekly leisure-time physical activity score was calculated according to the original scoring protocol. Smoking status was assessed by self-report and categorized as current smoker or non-smoker. Body weight and height were self-reported by participants and used to calculate BMI as weight (kg) divided by height squared (m^2^). Alcohol consumption was assessed based on self-reported intake of beer, wine, and spirits. Alcohol intake was expressed as grams of ethanol per week and calculated using the formula: grams of ethanol = volume (mL) × alcohol by volume × 0.789 (density of ethanol). Average alcohol contents of 5% for beer, 12% for wine, and 40% for spirits were assumed.

### 2.3. Psychological Distress Assessment

Psychological distress was assessed using the Depression Anxiety Stress Scales (DASS-21), a 21-item self-report instrument comprising three subscales (depression, anxiety, and stress), assessing the frequency of negative emotional states over the week prior to data collection [26]. Subscale scores were calculated by summing item responses and multiplying by two to obtain scores comparable to the original DASS-42. To reduce dimensionality and account for shared variance between subscales, subscale scores were standardized (z-scores) and averaged to obtain a composite index of psychological distress, which was re-standardized prior to inclusion in sensitivity analyses.

### 2.4. Dietary Supplement Assessment

Supplement use was assessed by self-report, with participants reporting the specific types of supplements used regularly. Due to the lack of detailed information on dosage, frequency, and limited information on supplement formulation, supplements were categorized into broad functional groups: multinutrient preparations, vitamins, minerals, fatty acids, biomass and plant-derived products (including algae, fungi, herbs, microbial biomass, and their derivatives), protein supplements, and other bioactive compounds (e.g., methylsulfonylmethane, hyaluronic acid). Multinutrient preparations included commercially available combinations of vitamins, minerals, and other bioactive compounds, ranging from simple multivitamin or multimineral formulations to more complex products containing additional components such as herbal extracts, probiotics, or immune-supporting compounds. These categories were used for descriptive analyses. In addition, supplement use was defined as the number of distinct supplement categories used per participant and examined in sensitivity analyses.

### 2.5. Blood Sampling, Freezing, and Storage

Sampling was conducted during the colder months (October 2020 to April 2021), with concurrent recruitment of both dietary groups to minimize potential seasonal variation in DNA damage parameters. Venous blood samples were collected by a trained medical professional in the morning after an overnight fast and avoidance of vigorous physical activity for at least 12 h, using ethylenediaminetetraacetic acid (EDTA) as the anticoagulant (Becton Dickinson, Franklin Lakes, NJ, USA). Whole blood aliquots were stored at −80 °C without cryoprotectants for up to one year prior to analysis. Before comet assay assessment, aliquots were rapidly thawed in a 37 °C water bath as previously described [27].

### 2.6. Alkaline Comet Assay

#### 2.6.1. Procedure

The alkaline comet assay was performed according to the protocol described by Collins et al. [28], in compliance with the Minimum Information for Reporting Comet Assay (MIRCA) guidelines [29]. After thawing at 37 °C, 5 µL of whole blood was mixed with 100 µL of 0.5% low-melting-point agarose (Sigma, St. Louis, MO, USA) and layered onto fully frosted microscope slides to form an agarose sandwich. The slides were immersed overnight at 4 °C in freshly prepared cold lysis solution containing 1% Triton X-100 (Sigma), 1% sodium N-lauroyl sarcosinate (Sigma), 2.5 M NaCl (Kemika, Zagreb, Croatia), 10% dimethyl sulfoxide (Kemika), 10 mM Tris-HCl (Sigma), and 100 mM disodium EDTA (Sigma) (pH 10). Following lysis and DNA unwinding, slides were transferred to alkaline electrophoresis buffer (1 mM disodium EDTA and 300 mM NaOH (Kemika), pH 13) for 20 min at 4 °C and subsequently electrophoresed for 20 min at 1 V/cm. After electrophoresis, the slides were neutralized with 0.4 M Tris-HCl buffer (pH 7.5) and stained with ethidium bromide (10 µg/mL; Sigma) before analysis.

All samples were processed under standardized conditions, with samples from both dietary groups distributed across analytical batches to minimize potential batch effects. All samples were processed using the same protocol, reagents, and instrumentation to support analytical consistency.

#### 2.6.2. Image Analysis

For each participant, a total of 100 randomly selected comets were analyzed on coded slides using an epifluorescence microscope (Zeiss, Oberkochen, Germany) equipped with appropriate filters at 250× magnification and the Comet Assay II (Perceptive Instruments Ltd., Haverhill, UK) image analysis software. Comets were evaluated from different, non-overlapping fields across the slide and were selected to avoid overlapping cells, debris, and damaged regions of the gel, including edges and areas without clearly defined comet heads. All slides were analyzed by a single trained operator under consistent conditions. DNA damage was quantified using the following comet descriptors: tail intensity (% tail DNA), tail length (µm), tail moment (calculated as the product of % tail DNA and tail length), and total comet area.

### 2.7. Statistical Analysis

Statistical analyses were performed using IBM SPSS Statistics (version 26; IBM Corp., Armonk, NY, USA). Comet assay descriptors were presented using Tukey-style boxplots with individual data points, generated in GraphPad Prism (version 9; GraphPad Software, San Diego, CA, USA). Participant characteristics were summarized using appropriate descriptive statistics.

The study sample size was calculated a priori using G*Power 3.1.9.7 (Heinrich-Heine-Universität Düsseldorf, Düsseldorf, Germany). The calculation was based on expected differences in comet assay tail intensity between dietary groups, using effect size estimates derived from a previous study conducted in a similar population [22]. Assuming a large effect size (*d* = 0.89), a minimum total sample size of *n* = 42 was required to achieve 80% power at a two-sided significance level of 0.05. The final sample size (*n* = 62) was above this estimate.

Tail intensity was selected as the primary outcome for inferential analyses based on its established role as the preferred descriptor of DNA strand breaks in the comet assay, in accordance with recommendations from the Organisation for Economic Co-operation and Development (OECD), as summarized in Collins et al. [28]. Associations between dietary pattern (vegan vs. omnivore) and tail intensity (% tail DNA) were examined using general linear models (GLM). An unadjusted model was followed by sequentially adjusted models including age, physical activity, BMI, and alcohol intake, selected a priori based on biological relevance [30]. Sensitivity analyses were performed by further adjusting the fully adjusted model separately for psychological distress (DASS-derived composite index) and supplement use (number of supplement categories per participant).

A secondary analysis among vegans examined the association between duration of adherence to a vegan diet and tail intensity, adjusted for age. Age was included as a covariate due to its potential confounding effect. Because a proportion of participants reported prior vegetarian adherence, total meat-free diet duration (including previous vegetarian diet) was examined as an alternative exposure measure in an age-adjusted model.

Model assumptions were assessed using standard residual diagnostics. Influential observations were evaluated using Cook’s distance, and multicollinearity was evaluated using variance inflation factors. The robustness of the fully adjusted model was further evaluated using bootstrap resampling (2000 iterations) with bias-corrected and accelerated (BCa) confidence intervals.

All tests were two-sided, with *p* < 0.05 considered statistically significant.

## 3. Results

### 3.1. Study Participant Characteristics

Characteristics of the study participants are summarized in Table 1. Sex and smoking status were identical due to matching, and mean age was similar across groups. The sample included a high proportion of women and individuals with higher education. Vegans had lower body mass index (BMI), higher physical activity scores, and lower alcohol consumption compared with omnivores.

The distribution of vegan diet duration was right-skewed. Thirteen of 31 vegans (41.9%) reported adherence to a lacto-ovo vegetarian diet before transitioning to a vegan diet. When considering total meat-free dietary exposure (including prior vegetarian adherence), the upper quartile of duration was 15.8 years. Ethical motivations were the most commonly reported reason for adopting a vegan diet (25/31, 80.6%), predominantly related to animal welfare and, less frequently, environmental sustainability.

**Figure A1:**
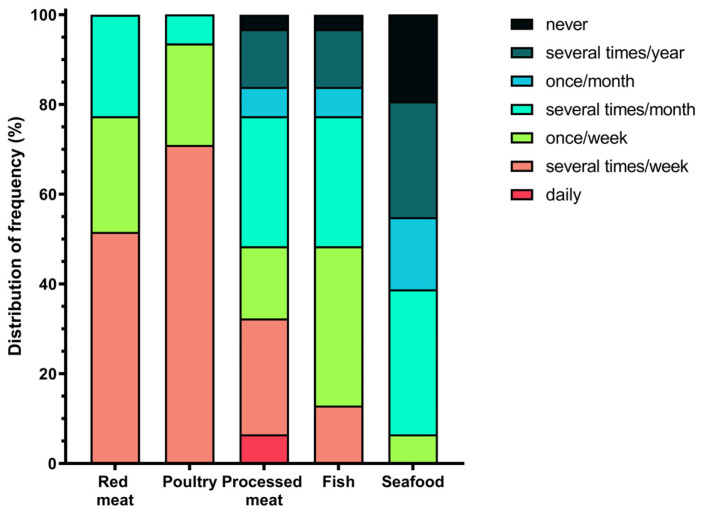
Distribution of consumption frequencies of meat, fish, and seafood among omnivorous participants assessed by the FFQ.

Omnivorous participants reported regular consumption of poultry, red meat, and processed meat, consistent with habitual mixed dietary patterns. Detailed consumption frequencies are shown in Appendix A (Figure A1).

Descriptive data on dietary supplement use and psychological distress are presented in Table 2. Overall dietary supplement use, particularly vitamin supplementation, was more frequently reported among vegans than omnivores. Other supplement categories were less frequently reported and showed no consistent pattern across the sample. The number of distinct supplement categories per participant had similar median values across groups; however, approximately 30% of vegans reported the use of three or more supplement categories, indicating a wider distribution compared with omnivores.

Psychological distress scores (DASS composite index) were similar between the two groups. Similarly, individual subscale scores for depression, anxiety, and stress showed no noticeable differences between vegans and omnivores.

### 3.2. Comet Assay Descriptor Distributions by Dietary Pattern

**Figure 1:**
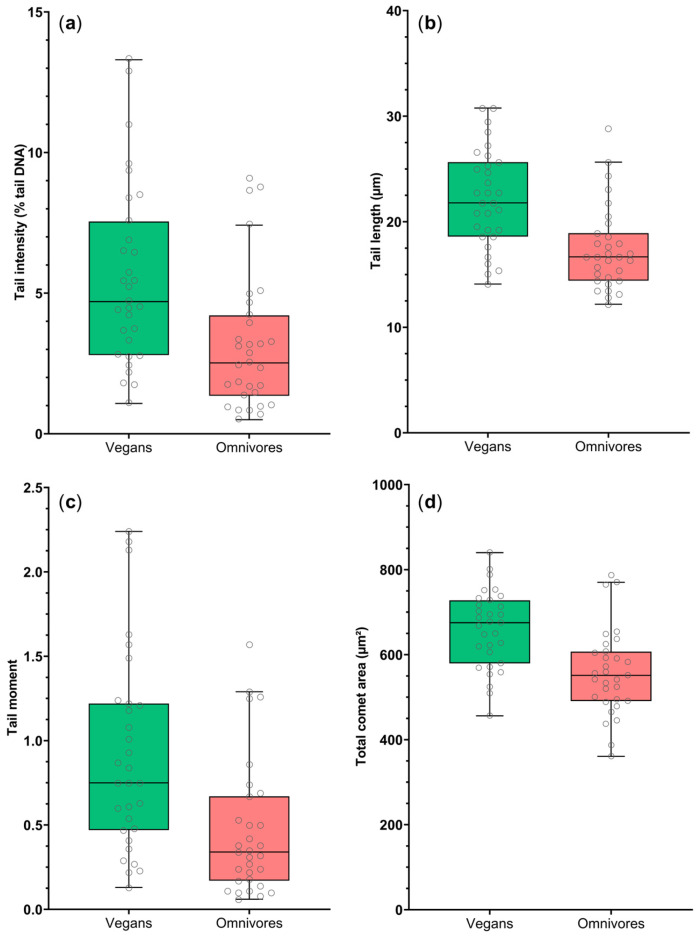
Distribution of comet assay descriptors by dietary pattern. (**a**) Tail intensity, (**b**) tail length, (**c**) tail moment, and (**d**) total comet area. Grey circles represent individual data points. Colors represent groups (green: vegans; red: omnivores).

Distributions of comet assay descriptors (tail intensity, tail length, tail moment, and total comet area) are shown in Figure 1. Median values were higher in vegans compared with omnivores, with a consistent upward shift in the distributions across all parameters. Median tail intensity was 4.7% tail DNA (IQR 2.8–7.6) in vegans and 2.5% tail DNA (IQR 1.4–4.2) in omnivores.

### 3.3. Association Between Dietary Patterns and DNA Damage

The association between dietary pattern and tail intensity (% tail DNA) was examined using sequentially adjusted general linear models (Table 3). In the unadjusted model, vegans exhibited higher tail intensity compared with omnivores (*B* = 2.39; 95% CI 0.94 to 3.84; *p* = 0.002). The association remained significant after adjustment for age and physical activity. In the fully adjusted model (Model 3), dietary pattern remained significantly associated with tail intensity (*B* = 1.98; 95% CI 0.19 to 3.76; *p* = 0.031; partial *η*^2^ = 0.081). Estimated marginal means of tail intensity were 5.35% (95% CI 4.19 to 6.50) among vegans and 3.37% (95% CI 2.22 to 4.53) among omnivores. None of the covariates were significantly associated with tail intensity. Model diagnostics did not indicate violations of assumptions or undue influence of individual observations, and the results were consistent in bootstrap analyses, with confidence intervals closely matching those obtained from the primary model (e.g., bootstrap 95% CI for dietary pattern: 0.42 to 3.38) and negligible bias observed.

Sensitivity analyses, including supplement use (number of supplement categories per participant, Model 4) and psychological distress (DASS composite index, Model 5), are presented in Appendix B (Table A1). The association between dietary pattern and tail intensity remained statistically significant after adjustment for supplement use. Supplement category count showed a tendency toward an inverse association with tail intensity (B = −0.70, *p* = 0.056), although this did not reach statistical significance. Inclusion of this variable increased the effect size of the dietary pattern (partial *η*^2^ from 0.081 to 0.107). Additional adjustment for psychological distress did not meaningfully alter the association between dietary pattern and tail intensity, and the distress score was not independently associated with the outcome.

### 3.4. Association Between Duration of Adherence to a Vegan Diet and DNA Damage

The association between duration of adherence to a vegan diet and tail intensity (% tail DNA) was examined using a general linear model adjusted for age (Table 4). Each additional year of vegan diet duration was associated with a 0.23% increase in tail intensity (*B* = 0.23; 95% CI 0.03 to 0.43; *p* = 0.026; partial *η*^2^ = 0.165). Age was not significantly associated with tail intensity in the adjusted model. When total meat-free diet duration (including prior vegetarian adherence) was examined as an alternative exposure measure, no significant association with tail intensity was observed (*B* = 0.10; 95% CI −0.05 to 0.25; *p* = 0.173).

## 4. Discussion

In this cross-sectional study, vegans exhibited higher levels of primary DNA damage, as reflected by higher comet assay tail intensity, compared with omnivores. The association remained significant after adjustment for age, physical activity, body mass index (BMI), and alcohol consumption. The observed association was further supported by the within-vegan analysis, in which longer duration of adherence to a vegan diet was positively associated with tail intensity, independent of age. In contrast, the total duration of meat-free diet adherence (including prior vegetarian diet) was not associated with tail intensity, suggesting that the relationship may differ depending on how dietary exposure is defined. Together, these findings suggest that adherence to a vegan diet and its duration are associated with biomarkers of primary DNA damage in this specific sample, although the underlying mechanisms cannot be determined from the present data. The comet assay primarily reflects transient DNA strand breaks and does not directly measure long-term genomic instability or mutation burden, which should be considered when interpreting these findings.

Although the four a priori-selected confounders (age, physical activity, BMI, and alcohol consumption) were not significantly associated with the outcome, this may reflect limited statistical power to detect smaller effects rather than a lack of biological relevance. Nonetheless, inclusion of these variables resulted in a substantial reduction in the effect size of dietary pattern (from 0.154 in the unadjusted model to 0.081 in the fully adjusted model), indicating that a considerable proportion of variance initially attributed to dietary pattern was accounted for by these covariates. It should be noted that the study was powered to detect a large effect size based on prior literature on vegetarians (rather than strict vegans), whereas adjustment for confounders resulted in a moderate effect size. While sufficient to detect the primary association, the modest sample size likely limited the precision of the estimates. This is reflected in the relatively wide confidence interval of the regression coefficient (0.19 to 3.76), indicating uncertainty in the magnitude of the difference in tail intensity between the groups.

An additional finding was the association between vegan diet duration and tail intensity, whereas total meat-free diet duration showed no such association. A total meat-free diet duration variable was constructed as the sum of current vegan and prior vegetarian adherence durations. The observed difference between vegan duration and total meat-free duration models may reflect the combined influence of exposure definition and sample composition. Specifically, individuals with short vegan duration but long prior vegetarian adherence may be classified as having substantially longer exposure in the total duration variable, potentially distorting the relationship with tail intensity. Additional imprecision may arise from the longer recall period required to estimate total meat-free duration. Further studies in larger samples are warranted to confirm the observed positive association between vegan diet duration and tail intensity.

We explored potential sources of residual confounding in sensitivity analyses, including psychological distress and supplement use. Neither the standardized composite DASS score nor a proxy for supplement diversity was significantly associated with tail intensity. Given that DASS-21 reflects recent psychological distress, these findings suggest that fluctuations in distress are unlikely to explain the observed differences in DNA damage between dietary groups. Notably, inclusion of the supplement proxy modestly increased the effect size of the dietary pattern variable. Although this adjustment strengthened the association, the interpretation of its biological significance is limited by the simplified measurement of supplement use and the non-causal nature of the study design. This pattern may indicate that supplement diversity captures aspects of dietary behavior not directly related to tail intensity, thereby clarifying the association between dietary pattern and the outcome. Overall, residual confounding cannot be excluded, as unmeasured variables, together with measurement error in the adjusted covariates, may still influence the observed association. In particular, unmeasured factors such as overall diet quality, sleep patterns, and other lifestyle-related behaviors may also contribute to the observed associations. All covariates were based on self-reported data and may be subject to reporting bias, particularly for socially desirable behaviors, such as higher physical activity and lower alcohol consumption.

Although standardized comet assay protocols were applied, residual methodological variability cannot be fully excluded and may have influenced the observed estimates. The absence of reference control samples limits the ability to assess inter-assay variability and to distinguish biological differences from potential batch-related shifts in measurement. While the balanced distribution of samples from both dietary groups across analytical batches reduces the likelihood of spurious group differences, such effects cannot be entirely excluded.

The use of snowball and voluntary response sampling may have influenced the composition of the sample, as snowball recruitment can lead to participants with shared social or behavioral characteristics, while voluntary participation may favor individuals who are more inclined to participate or have a greater interest in the study topic, potentially limiting representativeness in both dietary groups. In the present sample, these characteristics were reflected in a higher proportion of female participants and an overrepresentation of younger and highly educated adults. Within vegans, shared social networks may have contributed to similarities in motivations and behaviors among participants, which may also be reflected in dietary practices within this group. As suggested by Leitzmann [31], motivations for adopting vegetarian diets may influence dietary behaviors and nutritional adequacy. In the present study, the majority of vegan participants reported ethical concerns related to animal welfare as their primary motivation for adopting a vegan diet, which may be relevant for understanding dietary behaviors within this group. Dietary supplement use appeared to be common among vegans and may be associated with underlying motivations. Previous research has reported that supplementation practices may vary by motivation, with more frequent use observed among ethically motivated compared to health-motivated vegans [32]. Due to the lack of quantitative dietary data, imprecise measurement of supplement use, and overrepresentation of ethically motivated vegans, the present study cannot assess these relationships in detail within this sample. Similar considerations regarding generalizability apply to the omnivorous group. As omnivores are not defined by a distinct dietary identity to the same extent as vegans, snowball recruitment may have resulted in clustering based on other shared lifestyle or behavioral characteristics. These biases may introduce deviation from the general population in terms of dietary practices, health awareness, and lifestyle. To partially mitigate these effects, a multi-channel recruitment strategy was employed in both groups, including social networks and open-call invitations, which may have increased the diversity of the sample.

Evidence specifically linking a vegan diet with biomarkers of primary DNA damage in humans remains scarce. The findings of the present study align with a previous study conducted in a Croatian population sample that reported significantly higher comet assay tail intensity in vegetarians compared with non-vegetarians [22]. In contrast, studies conducted in other European populations generally reported no significant differences in primary DNA damage between vegans and omnivores [33] or vegetarians and omnivores [34,35,36]. Of note, studies that additionally assessed oxidative DNA damage suggested a tendency toward lower oxidative DNA damage in vegetarians [34,35,36]. Evidence from Asian populations remains inconsistent, with one study reporting lower primary DNA damage in vegetarians and another observing no significant difference [37,38]. More broadly, investigations using other cytogenetic biomarkers of genomic instability, including micronucleus frequency and sister chromatid exchanges, have likewise produced heterogeneous results. Verhagen et al. [33] observed no significant differences in micronucleus frequency between vegans and omnivores, consistent with their findings for primary DNA damage. Similarly, members of the Seventh-day Adventist community exhibited lower sister chromatid exchange frequencies compared with matched controls from the general population, although no differences were observed between vegetarians and omnivores within the Adventist group [39]. Fenech and Rinaldi [40] likewise reported no consistent differences in micronucleus frequency between vegetarians and non-vegetarians, with results varying across both age and sex strata. Differences in study populations, age distribution, dietary quality, and control of potential confounders may partly explain these discrepancies.

Although plant-based diets are generally characterized by higher intake of phytochemical-rich foods, which have been associated with reduced DNA strand breaks and oxidative DNA damage in human intervention and experimental studies [41,42,43], the present study did not include quantitative assessment of dietary intake or nutrient status, limiting the ability to evaluate such effects. Conversely, vegetarian and vegan dietary patterns may be associated with lower intake or bioavailability of certain micronutrients involved in DNA synthesis and repair, including vitamin B_12_, vitamin D, and iron [7,44,45,46]; however, this could not be assessed in the current study.

Overall, these considerations highlight the complexity of dietary exposures and the limitations of explaining underlying biological mechanisms in the absence of quantitative dietary data. Future studies should incorporate detailed dietary assessment, including adequacy of macro- and micronutrient intake, as well as overall diet quality, along with biochemical markers of nutritional status, to better characterize the relationship between dietary patterns and DNA damage.

Several limitations should be considered when interpreting these findings. First, the cross-sectional design limits causal inference, and the directionality of the observed associations cannot be established; therefore, reverse causation cannot be excluded. Second, participants were recruited using non-probability sampling methods, which may introduce selection bias and limit the representativeness of both dietary groups. Third, the relatively modest sample size may limit the precision of the estimated associations. Fourth, a quantitative assessment of dietary intake was not performed, limiting the ability to evaluate nutrient intake and overall diet quality. Fifth, residual confounding by unmeasured factors, including diet quality and other lifestyle-related variables, cannot be excluded. Finally, the absence of dedicated comet assay control samples limits the ability to formally assess assay variability.

## 5. Conclusions

In this study, higher levels of comet tail intensity were observed in peripheral blood cells of vegans compared with omnivores; however, these findings should be interpreted with caution given limited external validity, modest sample size, and the cross-sectional design, which precludes causal inference. The observed association may be influenced by a complex interplay of diet quality, dietary behaviors, and other lifestyle-related factors. Overall, interpretation of the findings is constrained by the complexity of dietary exposures and the limited capacity of the present dataset to evaluate diet-related factors of inter-individual variability in comet assay tail intensity, particularly in the absence of quantitative intake data and biochemical markers of nutritional status. Further studies incorporating detailed nutrient profiling and comprehensive assessments of diet quality are warranted to clarify potential mechanistic pathways linking dietary patterns with primary DNA damage.

## Figures and Tables

**Table 1:** General characteristics of the study participants.

Characteristic	Vegans (*n* = 31)	Omnivores (*n* = 31)
Age (y), mean ± SD	33.2 ± 9.4	32.7 ± 8.7
Female sex, *n* (%)	21 (67.7%)	21 (67.7%)
Smokers, *n* (%)	11 (35.5%)	11 (35.5%)
BMI (kg/m^2^), mean ± SD	21.1 ± 1.9	22.9 ± 2.2
Alcohol consumption (g/week), median (IQR)	0.0 (0.0–27.3)	22.5 (0.0–67.5)
Physical activity score, median (IQR)	35 (28.5–39.5)	21 (12.5–39.0)
Higher education, *n* (%)	23 (74.2%)	26 (83.9%)
Vegan diet duration (y), median (IQR)	4.5 (2.3–10.0)	−
Total meat-free diet duration (y), median (IQR)	5.5 (3.5–15.8)	−

Data are presented as mean ± SD, median (IQR), or *n* (%), as appropriate. Higher education was defined as completion of university-level education (bachelor’s degree or higher). BMI, body mass index; −, not applicable.

**Table 2:** Dietary supplement use and psychological distress across dietary patterns.

Characteristic	Vegans (*n* = 31)	Omnivores (*n* = 31)
Supplement use		
Vitamins, *n* (%)	24 (77.4%)	11 (35.5%)
Minerals, *n* (%)	7 (22.6%)	9 (29.0%)
Multinutrient preparations, *n* (%)	4 (12.9%)	5 (16.1%)
Fatty acids, *n* (%)	6 (19.4%)	2 (6.5%)
Biomass and plant-derived products, *n* (%)	6 (19.4%)	1 (3.2%)
Protein supplements, *n* (%)	5 (16.1%)	2 (6.5%)
Other, *n* (%)	3 (9.7%)	0 (0.0%)
Any supplement use, *n* (%)	27 (87.1%)	17 (54.8%)
Supplement category count, median (IQR)	1 (1–3)	1 (0–2)
Psychological distress (DASS-21)		
Depression score, median (IQR)	4 (2–8)	4 (1–6)
Anxiety score, median (IQR)	4 (2–6)	2 (0–8)
Stress score, median (IQR)	12 (6–14)	10 (6–17)
DASS composite index (z-score), mean ± SD	−0.01 ± 0.91	0.01 ± 1.10

Data are presented as *n* (%), median (IQR), or mean ± SD, as appropriate. DASS, Depression Anxiety Stress Scales; DASS-21 scores were multiplied by 2 to obtain values comparable to the DASS-42.

**Table 3:** Association between dietary pattern and tail intensity (% tail DNA) in sequentially adjusted general linear models (*n* = 62).

	Model 1		Model 2		Model 3	
Predictor	*B* (95% CI)	*p*	*B* (95% CI)	*p*	*B* (95% CI)	*p*
Dietary pattern (vegan vs. omnivore)	2.39 (0.94, 3.84)	0.002	2.48 (0.96, 4.00)	0.002	1.98 (0.19, 3.76)	0.031
Age (y)	−	−	0.03 (−0.06, 0.11)	0.507	0.03 (−0.05, 0.11)	0.477
Physical activity score	−	−	−0.01 (−0.05, 0.03)	0.600	−0.01 (−0.05, 0.03)	0.767
BMI (kg/m^2^)	−	−	−	−	−0.12 (−0.49, 0.26)	0.532
Alcohol (g/week)	−	−	−	−	−0.01 (−0.03, 0.01)	0.232
Partial *η*^2^	0.154		0.156		0.081	
Model *R*^2^	0.154		0.164		0.190	

Values represent unstandardized regression coefficients (*B*) with 95% confidence intervals and *p*-values. Partial *η*^2^ represents the effect size for the dietary pattern in each model. Dietary pattern was coded with omnivores as the reference category. Model 1: unadjusted. Model 2: adjusted for age and physical activity. Model 3: adjusted for age, physical activity, BMI, and alcohol consumption. −, not applicable.

**Table 4:** Association between vegan diet duration and % tail DNA among vegans (*n* = 31).

Predictor	*B* (95% CI)	*p*
Vegan diet duration (y)	0.23 (0.03, 0.43)	0.026
Age (y)	−0.13 (−0.29, 0.04)	0.130
Partial *η*^2^	0.165	
Model *R*^2^	0.165	

Values represent unstandardized regression coefficients (*B*) with 95% confidence intervals and *p*-values. Partial *η*^2^ represents the effect size for vegan diet duration in the adjusted model. The model was adjusted for age.

## Data Availability

The original contributions presented in this study are included in the article. Further inquiries can be directed to the corresponding author.

## Abbreviations

The following abbreviations are used in this manuscript:

BMI	Body mass index
CI	Confidence interval
DASS-21	Depression Anxiety Stress Scales
EDTA	Ethylenediaminetetraacetic acid
FFQ	Food frequency questionnaire
GLM	General linear model
GLTEQ	Godin Leisure-Time Exercise Questionnaire
hs-CRP	High-sensitivity C-reactive protein
IQR	Interquartile range
MIRCA	Minimum Information for Reporting Comet Assay
OECD	Organisation for Economic Co-operation and Development
SD	Standard deviation

## Appendix B

**Table A1:** Association between dietary pattern and tail intensity (% tail DNA) in general linear models with additional adjustments (*n* = 62).

	Model 4		Model 5	
Predictor	*B* (95% CI)	*p*	*B* (95% CI)	*p*
Dietary pattern (vegan vs. omnivore)	2.26 (0.49, 4.03)	0.013	1.94 (0.12, 3.75)	0.037
Age (y)	0.07 (−0.02, 0.17)	0.117	0.03 (−0.06, 0.11)	0.470
Physical activity score	0.00 (−0.04, 0.04)	0.852	−0.01 (−0.05, 0.03)	0.751
BMI (kg/m^2^)	−0.17 (−0.54, 0.20)	0.350	−0.14 (−0.53, 0.26)	0.486
Alcohol (g/week)	−0.02 (−0.04, 0.01)	0.140	−0.01 (−0.03, 0.01)	0.222
Supplement category count	−0.70 (−1.41, 0.02)	0.056	−	−
DASS composite index	−	−	−0.15 (−0.93, 0.64)	0.712
Partial *η*^2^	0.107		0.077	
Model *R*^2^	0.242		0.192	

Values represent unstandardized regression coefficients (*B*) with 95% confidence intervals and *p*-values. Partial *η*^2^ represents the effect size for the dietary pattern in each model. Dietary pattern was coded with omnivores as the reference category. Model 4: Model 3 adjusted for supplement category count. Model 5: Model 3 adjusted for DASS composite index. −, not applicable.
